# The Effect of Acupuncture on the Motor Function and White Matter Microstructure in Ischemic Stroke Patients

**DOI:** 10.1155/2015/164792

**Published:** 2015-10-20

**Authors:** Yongxin Li, Ya Wang, Heye Zhang, Ping Wu, Wenhua Huang

**Affiliations:** ^1^Institute of Clinical Anatomy, School of Basic Medical Sciences, Southern Medical University, Guangzhou 510515, China; ^2^Shenzhen Institutes of Advanced Technology, Chinese Academy of Sciences, Shenzhen 510855, China; ^3^The 3rd Teaching Hospital, Chengdu University of Traditional Chinese Medicine, Chengdu, Sichuan 610075, China

## Abstract

Evidence shows that ischemic stroke can induce brain structural reorganization. Acupuncture is advised as an adjunct to mainstream rehabilitation after stroke. However, the effectiveness of acupuncture is inconsistent among previous studies. Fourteen ischemic patients were collected and divided into two groups: conventional treatment group (CG) and acupuncture treatment group (AG). The results of a Fugl-Meyer Assessment (FMA) and diffusion tensor imaging were collected before and after treatment. The AG exhibited a higher improvement in FMA than the CG. Repeated measures analysis of variance on diffusion data only found a significant main effect for scanning time point in all diffusion indices. In each group, a postpair *t*-test revealed that diffusion indices values were changed significantly after treatment intervention in the body of the corpus callosum and bilateral corticospinal tracts, the inferior longitudinal fasciculus, the inferior frontooccipital fasciculus, the superior longitudinal fasciculus, the forceps minor, the cingulum gyrus, and the thalamic radiation. However, there was no significant difference in the diffusion indices between the two groups. In conclusion, acupuncture had a better behavioral score than traditional medicine treatment. However, acupuncture did not significantly change WM in the AG compared to the CG as expected within one month after the intervention.

## 1. Introduction

Recently, stroke has become the focus of our attention due to its high mortality and adult disability rates [1]. Many ischemic stroke patients experience a poor prognosis with neurological and motor function impairment, such as spastic paralysis, which greatly lowers the quality of life of stroke survivors. Various therapies have been used to conquer the sequela of stroke, including medication, rehabilitation, and surgery, but the effectiveness of these treatments remains unsatisfactory. Thus, improvement of stroke prognosis effectively needs urgent attention.

Acupuncture, a traditional Chinese medicine, has gained international attention with respect to its safety and efficacy as an adjunctive therapy for improving behavior after ischemic stroke [2–4]. Although several randomized controlled clinical trials did not achieve their expected outcomes, an increasing number of studies have confirmed that acupuncture has a positive modulatory effect on poststroke rehabilitation [5–9]. Currently, there are many different hypotheses on the mechanism of acupuncture in the motor recovery of ischemic stroke patients. One study showed that there were different cerebrovascular responses between normal individuals and stroke patients after acupuncture [10], confirming that acupuncture stimulation activates perilesional or use-dependent reorganization of ischemic sites through improvement in regional cerebral blood flow (CBF). The activation in the hypoperfused zone is consistent with the perilesional neuronal plasticity if adequate rehabilitative training is performed [11]. Many other studies also confirmed that acupuncture plays an important role in the improvement in regional CBF [12–14]. One study from Zhang, who performed a microarray analysis of stroke patients, showed that acupuncture after intervention regulates gene expression [15]. This regulation of gene expression may be associated with the recovery after stroke, which is a possibility that is currently under study. Acupuncture also has neuroprotective effects, which are related to the mechanism of acupuncture [16–19]. However, few studies have investigated changes in the white matter tract after acupuncture in ischemic stroke patients. A previous animal study found that acupuncture treatment improved motor function and the relative fractional anisotropy (FA) value at the edge of the ischemic lesions of rats [9]. In human, previous studies found that there is a positive correlation between the FA values of the motor tracts and the recovery of motor function in stroke patients [20, 21]. Based on these findings, we hypothesized that acupuncture would lead to an enhancement of patients' motor function and changes in their white matter (WM) microstructure.

Therefore, the purpose of the present study was to examine the effects of acupuncture on motor function and brain WM microstructure in ischemic stroke patients. Before and after four weeks of therapy, we performed diffusion tensor imaging (DTI) and a Fugl-Meyer Assessment (FMA) on two groups. DTI is based on measuring water molecular diffusion along axons within every voxel in an image, which reveals individual tissue diffusion characteristics in ischemic stroke patients [22–24]. Studies have confirmed the reliability of tract-based DTI analysis approaches in evaluating the integrity of WM fibers in well-recovered individuals with chronic stroke and healthy participants [25, 26]. Tract-based approaches provide us an appropriate marker rather than a redundant indicator of the microstructure properties of WM tracts. The FMA scale is a disease-specific objective impairment index designed specifically as an evaluative measure for the assessment of recovery in the poststroke hemiplegic patients and is a highly accepted measure [27]. This scale is currently being used with increasing frequency [28, 29]. Thus, we chose to use the tract-based spatial statistics (TBSS) analysis method and the FMA scale to evaluate our hypothesis that acupuncture could affect WM microstructure and change WM tracts in association with motor score.

## 2. Materials and Methods

### 2.1. Subjects

**Figure 1:**
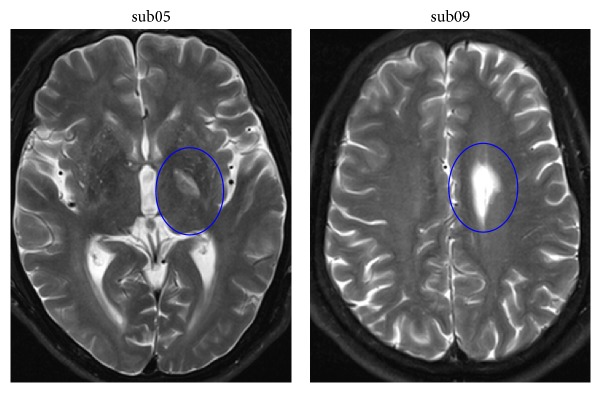
The example of lesion's location and volume in two patients.

Fourteen first-ever stroke patients (see Table 1 for clinical details) with unimanual motor deficits due to subcortical ischemic lesions were recruited from the Department of Neurology of the First Affiliated Hospital of the Chengdu University of Traditional Chinese Medicine, China. None of the patients had a history of neurological or psychiatric disorders, and no patients presented with aphasia, neglect, or dementia. No patient had undergone any other experimental therapy at the time of enrollment. All of the patients were scanned using DTI at two time points: before and after one month of clinical treatment. An example of lesion location and size can be seen in Figure 1.

The study protocol was approved by the Ethics Committee of the Chengdu University of Traditional Chinese Medicine. Every participant was informed of the purpose and procedure of this study. Informed consent was obtained from each participant prior to the study.

### 2.2. Treatment and Clinical Assessments

Seven stroke patients were given a conventional treatment of antiplatelet aggregation drugs to improve blood circulation (conventional treatment group, CG). Other seven patients were given acupuncture and conventional treatment (acupuncture treatment group, AG). Acupuncture was performed at the Baihui (GV20), Fengchi (GB20, bilateral), Xuanzhong (GB39, bilateral), Quchi (LI11 bilateral), Hegu (LI4, bilateral), Zusanli (ST36, bilateral), and Sanyinjiao (SP6, bilateral) acupoints. Based on the theory of Chinese medicine, these acupoints are often used in the treatment of motor dysfunction after stroke. All acupuncture procedures were performed by experienced and licensed acupuncturists, with Baihui and Fengchi forward flat spines 1.0–1.5 inch, obliquely to the tip of the nose direction of the wind pool. The remaining acupoints were down to levels of 0.8–1.5 inch. The twisting angle was less than 90 degrees. Treatments were conducted for thirty minutes per day, 5 days per week, one week per course, over four continuous courses of treatment. During the acupuncture treatment process, the dose of medication was adjusted by clinicians according to the patients' conditions.

The clinical performances were assessed before and after treatment to quantify the motor skill and the severity of the neurological functional deficits in the stroke patients using the FMA. The FMA is a disease-specific objective impairment index [27]. Higher scores denote a milder impairment of motor function. A 2-factor, repeated-measures ANOVA with the factors group (AG versus CG) and time point (before versus after) on the FMA was calculated. For each patient, improvement in clinical performance was calculated using the following formula: abs (after − before)/before. These improvements were compared between both groups in a two-sample* t*-test analysis.

### 2.3. Image Acquisition

Imaging data were collected using an 8-channel head coil on a 3T Siemens scanner (MAGNETOM Trio Tim, Siemens, Germany) at the West China Hospital MRI Center, Chengdu, China. The DTI protocol involved using a spin echo planar image sequence with the following parameters: TR/TE = 6800/93 ms, FOV = 240 × 240 mm^2^, 50 axial slices, slice thickness = 3 mm, and in-plane resolution = 1.875 × 1.875 mm^2^. Diffusion weighing was isotropically distributed along 30 directions (*b* = 1000 s/mm^2^). The acquisition of the diffusion-weighted images was performed in blocks of 2 images with no diffusion weighting (*b* = 0 s/mm^2^). The images that were not diffusion-weighted served as an anatomical reference for motion correction. Foam cushions were used to reduce head translation movement and rotation. All acquisitions were visually inspected for imaging artifacts. None of the participants were excluded on this basis.

### 2.4. Imaging Processing and Statistical Analysis

#### 2.4.1. TBSS Analysis

The DTI data were analyzed using the FMRIB Software Library (University of Oxford, FSL v5.0.1, http://www.fmrib.ox.ac.uk/fsl/). Standard processing steps were used, as described in detail previously [30]. First, eddy currents and head motion correction were carried out using affine registration to the first no diffusion-weighted image [31]. The data were then skull-stripped using FMRIB's Brain Extraction Tool (BET v2.1) [32]. Subsequently, FMRIB's Diffusion Toolbox (FDT v3.0) was used to fit the diffusion tensor and calculate the eigenvector and eigenvalue (*λ*1, *λ*2, and *λ*3) at each voxel [33]. Diffusion measures commonly used to characterize microstructural features of WM include FA, axial diffusivity (AD, corresponding to *λ*1), mean diffusivity (MD, corresponding to (*λ*1 + *λ*2 + *λ*3)/3), and radial diffusivity (RD, corresponding to (*λ*2 + *λ*3)/2).

TBSS (part of FSL [34]) was used to perform voxelwise analyses of FA between the AG and the CG patients. The TBSS procedure has been described in detail elsewhere [35]. Briefly, all subject's FA images were first aligned into a standard brain space using FMRIB's Nonlinear Image Registration Tool. Next, the mean FA images were created and thinned using a projection technique to create a mean FA skeleton, which represents the centers of major tracts common to all subjects. A threshold of 0.2 was used for the creation of the mean skeleton. Each subject's aligned FA images were then projected onto this skeleton. Finally, the projection data were fed into voxelwise general linear modeling cross-subject statistics. AD, MD, and RD skeletons were constructed with skeleton-projection parameters from the FA skeleton procedure, using tbss_non_FA procedure provided in FSL. The John Hopkins University (JHU) ICBM-DTI-81 white matter atlas was used for labeling the regions showing significant differences between groups [36].

To explore the possible local alteration of WM tracts, permutation-based nonparametric inference (*n* = 5000) was adopted to perform statistical analyses on FA, AD, MD, and RD. A repeated-measures analysis of variance (2 groups × 2 time points ANOVA) was used to evaluate the effects of group and time point on the diffusion indices. Significant interactions and main effects were followed up with* t*-tests for pairwise comparisons. The statistical threshold was established as *p*_FWE_ < 0.05 with multiple comparison correction using threshold-free cluster enhancement [37]. Then, we tested for relationships between the rate of WM microstructural changes and the treatment-related FMA improvement in each group. The significance threshold for the correlations was set at *p* < 0.05. To minimize their possible impact on the findings, gender, lesion size, and age were used as covariates of no interest in all the statistical analyses above.

## 3. Results

### 3.1. Behavioral Measures

Information on all subjects can be found in Table 1. ANOVA analyses revealed a main effect of time point on FMA score (*F*_1,12 _= 24.89, *p* < 0.001), demonstrating that the FMA score was enhanced significantly for both treatment groups (AC:* t* = 7.89, *p* < 0.001; CG:* t* = 7.77, *p* < 0.001). In contrast the main effect of group on the FMA score was not significant (*F*_1,12 _= 0.96, *p* = 0.337). There was no significant group by time point interaction (*F*_1,12 _= 2.87, *p* = 0.10). A two-sample* t*-test on the difference in FMA improvement showed a significant difference in improvement between groups (AC: 0.13 ± 0.05, CG: 0.06 ± 0.02;* t*(12) = 3.25, *p* = 0.007). Then, age, gender, lesion size, and age were considered as covariates of no interest in the analyses. The difference in FMA improvement still showed a significant difference in improvement between groups. The AG exhibited a higher improvement in FMA than the CG.

### 3.2. Voxelwise Statistics of FA, AD, MD, and RD

**Figure 2:**
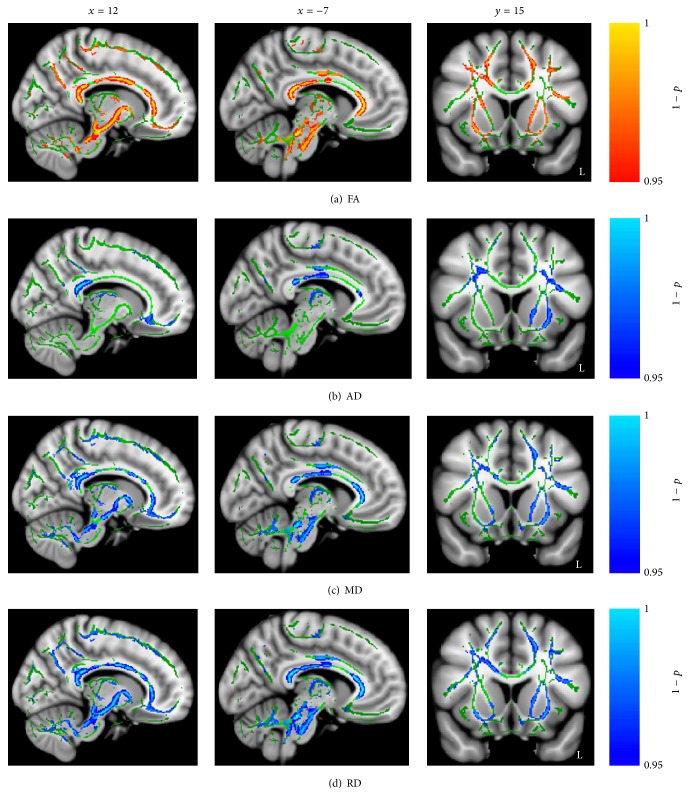
Significant main effect of time point in diffusion indices. (a) Regions of enhanced FA in stroke patients with treatment intervention. (b–d) Regions of reduced AD, MD, and RD in stroke patients with treatment intervention. Shown are cluster in the CC, bilateral CST, ILF, IFOF, SLF, forceps minor, cingulate gyrus, and thalamic radiation.

Repeated-measures ANOVA analysis revealed a significant main effect of time point in all diffusion indices. With treatment intervention, an increased FA and decreased AD, MD, and RD were found in several brain regions (*p*_FWE_ < 0.05). These regions included the corpus callosum (CC), the bilateral corticospinal tracts (CST), the inferior longitudinal fasciculus (ILF), the inferior frontooccipital fasciculus (IFOF), the superior longitudinal fasciculus (SLF), the forceps minor, the cingulate gyrus, and the thalamic radiation (Figure 2). There was neither a significant main effect of group nor a significant group-by-time point interaction in all diffusion indices.

**Figure 3:**
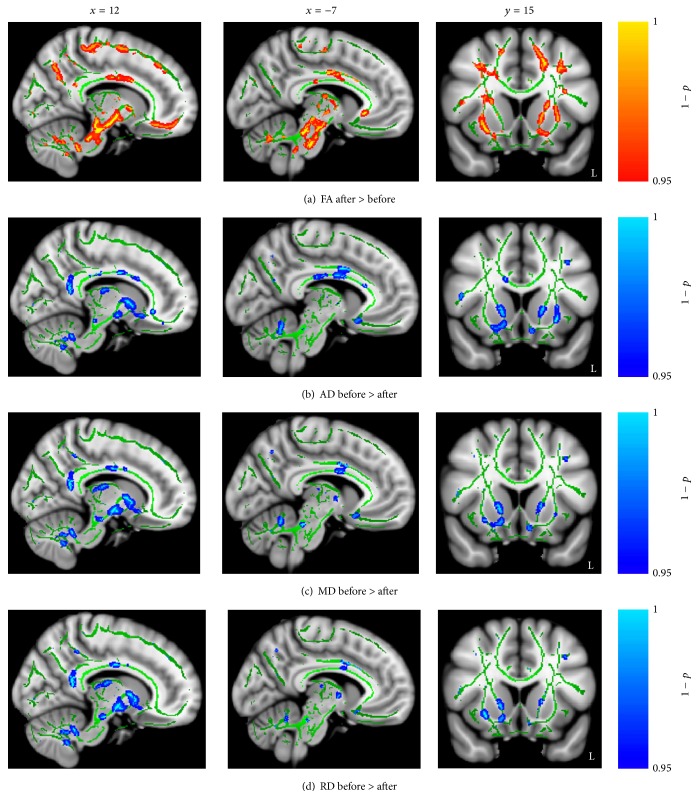
Significant changes in diffusion indices with theacupuncture treatment effect. (a) Regions of enhanced FA in stroke patients with acupuncture treatment. (b–d) Regions of reduced AD, MD, and RD in stroke patients with acupuncture treatment. Shown are cluster in the body of CC, CST, ILF, IFOF, forceps minor, cingulate gyrus, and anterior thalamic radiation.

**Figure 4:**
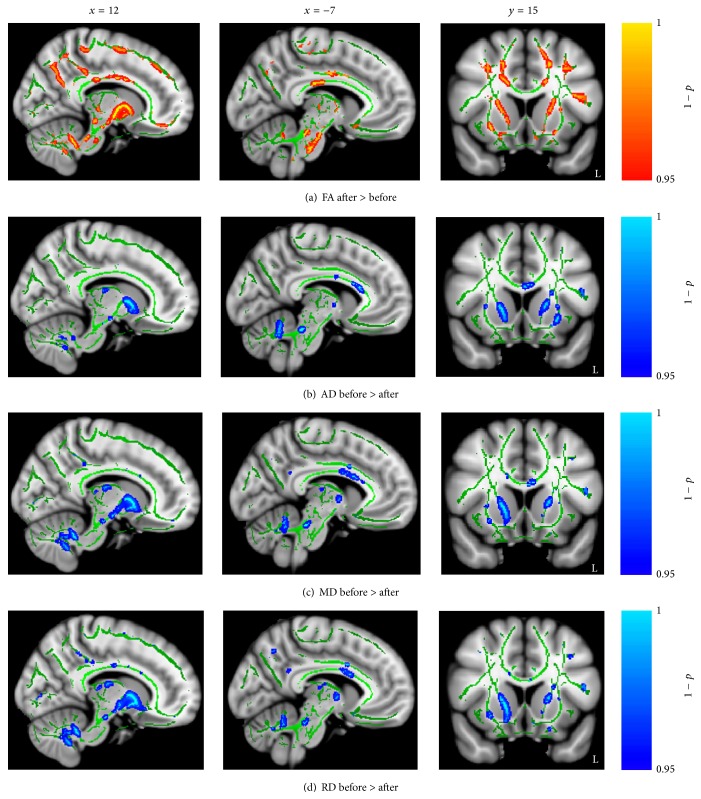
Significant changes in diffusion indices with theconventional treatment effect. (a) Regions of enhanced FA in stroke patients with conventional treatment. (b–d) Regions of reduced AD, MD, and RD in stroke patients with conventional treatment. Shown are cluster in the body of CC, CST, ILF, IFOF, forceps minor, cingulate gyrus, and anterior thalamic radiation.

A postpair *t*-test revealed that FA values were increased significantly (*p*_uncorrected_ < 0.05) after acupuncture treatment intervention in the body of the CC, the bilateral CST, the ILF, the IFOF, the forceps minor, the cingulate gyrus, and the anterior thalamic radiation (Figure 3(a)). AD, MD, and RD values were decreased significantly (*p*_uncorrected_ < 0.05) with acupuncture treatment intervention in the body of the CC, the right CST, the bilateral ILF, the IFOF, the cingulate gyrus, and the anterior thalamic radiation (Figures 3(b)–3(d)). Similarly increased FA and decreased direction diffusion indices were found in the CG after conventional treatment intervention (Figure 4).

Then, the diffusion indices values were extracted from the areas showing significant changes after treatment intervention. The rate of WM microstructural changes was calculated with the following formula: abs (after − before)/before. Correlation analyses revealed that there were no significant correlations between the rate of WM microstructural changes and the treatment-related FMA improvement in each group.

## 4. Discussion

In this study, we examine the treatment effects of acupuncture on stroke patients' motor function and WM microstructure. Different treatment interventions were used, and the treatment effects were compared for different methods. There were several main findings. First, acupuncture led to a higher improvement in FMA than conventional treatment. Second, patients in the AG did not show differences in the diffusivity pattern in WM tracts compared with the CG using the TBSS method. With treatment intervention, increased FA and decreased direction diffusion indices were found in both groups, which indicated the presence of changes in WM after stroke [38–40]. However, acupuncture did not lead to significant differences in the diffusion indices between the two groups. Third, there was no significant correlation between the rate of WM microstructure changes and the treatment-related FMA improvement in each group.

The behavioral results of the FMA were different for ischemic patients between the two treatment methods. The findings imply that acupuncture could improve motor function in ischemic stroke patients, which is consistent with recent studies [41, 42]. The neuroimaging results showed that increased FA and decreased direction diffusion indices were found in both groups with treatment intervention. The changed regions are similar to each other. FA is highly sensitive to changes in WM microstructure, as measured by water anisotropy in neural fibers [43]. AD, RD, and MD are served as the multiple diffusion tensor measures to maximize the specificity as a supplement of FA [44]. Most studies show that higher FA values are associated with an improvement in functional outcomes and that decreased FA values are associated with neurological or psychiatric disorders [39, 45–47]. In our study, increased FA and decreased AD, MD, and RD indicated that there was some improvement in the integrity of WM tracts in each group after treatment intervention. However, there were no significant differences in the changes of WM microstructures measured by TBSS between AG and CG.

These results between groups exceeded our expectations. Different acupuncture acupoints have discordant effects on patient rehabilitation. We chose appropriate acupoints that have been shown to improve limb motor function [48, 49]. Stimulating the Quchi and Hegu acupoints could change regional cerebral blood flow in stable somatosensory stroke patients [49]. A review showed that Baihui-based scalp acupuncture resulted in a large improvement in infarct volume and neurological function score [48]. Thus, we chose the Quchi, Hegu, Baihui, and Fengchi acupoints, which are associated with motor function, hoping for better patient outcomes. The behavioral results in the present study were as expected, but the neuroimaging results were not. This result may have been due to the specificity of the acupuncture treatment. Acupuncture is advised as an adjunct to mainstream rehabilitation after stroke. Its effects on brain activation and cerebral blood flow have been studied previously [50, 51]. Previous studies have demonstrated a stronger activation in the ante meridiem condition than in the postmeridiem condition in both healthy and stroke subjects treated with acupuncture [51]. The duration time of acupuncture plays an important role in the brain activation patterns. Our research here also showed that the activated regions were different. Another study found that a 60-second duration yielded a better outcome with rapid cerebral blood flow, better recovery of neurological function, and small cerebral infarct volume [50]. In the present study, all acupuncture procedures were performed by an experienced and licensed acupuncturist. Acupuncture was performed for two hours per day for each patient in the AG. Although we controlled the acupuncture needle-retention time, the time of the acupuncture procedure was not strictly controlled, which might have affected the neuroimaging results.

Furthermore, a previous study found that the treatment of stroke with acupuncture should be a long-term process. Patients' sensory, motor, and functional scores improved significantly until 2 years after injury with acupuncture treatment in one study [52]. In the present study, with a one-month acupuncture intervention, the motor function of patients in the AG was significantly improved as compared to the CG. Such discrepancies between the results of the current study and those of Hegyi and Szigeti may be due to differences in the therapeutic methods. Hegyi and Szigeti investigated the effects of treatment using Yamamoto new scalp acupuncture, whereas the current study examined the treatment effects using traditional Chinese acupuncture in combination with western medicine.

Our analysis did not find a correlation between the WM microstructure and motor function in each group. This result is not consistent with a previous study, which found a positive correlation between the FA values of motor tracts and the recovery of motor function in stroke patients [20]. Another study also showed that grip strength correlated with the integrity of the CSTs originating from primary motor and dorsal premotor cortices [21]. The potential mechanism is that the CC or CST connects the motor-related cortices, and the higher cortices then modulate motor function through a modulation of the CC or CST. The protective and reorganizational effects of acupuncture on WM tracts were not as we expected in our study. The reason for such discrepancies may be that the drug treatment altered the effects of acupuncture on WM tracts in acute-stage ischemic stroke patients. Other confounding factors might have affected our imaging results, such as the inconsistent brain injury sites. In addition, we completed our study using a small sample with heterogeneous lesion sites and a wide range of lesion ages. Additionally, the AG group was older than the CG group. These differences might have affected our results. Future research with a larger cohort may identify the consistent changes associated with treatment.

## 5. Conclusion

The current study demonstrated that there was an improvement in motor function after acupuncture treatment compared to conventional treatment. In each group, neuroimaging results showed that diffusion indices in WM tracts were significantly enhanced one month after treatment. However, no differences in the diffusivity pattern in WM tracts were found between the groups, compared to the CG using the TBSS method. We did not evaluate the correlation between the rate of WM microstructure changes measured by TBSS and the treatment-related FMA improvement in the two groups. Some limitations in our study that were difficult to control might have led to undesirable outcomes. Further study is necessary.

## Figures and Tables

**Table 1:** Demographic and imaging data.

Patient number	Age (yr)	Gender	Dominant hand	Affected hand	Site of lesion	Lesion volume (mm^3^)	Lesion age (days)	FMA1	FMA2
AG									
1	69	F	R	R	L Pons/CS	350	135	90	96
2	74	F	R	R	L/R TH	420	22	74	90
3	67	M	R	R	L BG	1180	33	80	93
4	78	M	R	R	L BG	3120	21	84	95
5	81	M	R	R	L BG/CS	1350	22	83	92
6	62	M	R	R	L BG/CS	200	36	82	95
7	71	F	R	L	R BG	2240	32	88	95
CG									
8	49	M	R	R	L BG	380	56	85	93
9	54	F	R	R	L BG	1190	45	80	85
10	73	M	R	R	L BG	540	26	84	91
11	73	M	R	R	L BG	1130	132	74	90
12	63	M	R	R	L TH	290	21	82	85
13	54	F	R	R	L BG	280	23	86	92
14	43	F	R	R	L CN	450	25	92	96

BG: basal ganglia; CN: caudate nucleus; CS: centrum semiovale; F: female; FMA: Fugl-Meyer Motor Assessment scale; L: left; M: male; R: right; TH: thalamus.
