# Erratum to “The Effect of Acupuncture on the Motor Function and White Matter Microstructure in Ischemic Stroke Patients”

**DOI:** 10.1155/2016/4259430

**Published:** 2016-05-24

**Authors:** Yongxin Li, Ya Wang, Heye Zhang, Ping Wu, Wenhua Huang

**Affiliations:** ^1^Institute of Clinical Anatomy, School of Basic Medical Sciences, Southern Medical University, Guangzhou 510515, China; ^2^Shenzhen Institutes of Advanced Technology, Chinese Academy of Sciences, Shenzhen 510855, China; ^3^The 3rd Teaching Hospital, Chengdu University of Traditional Chinese Medicine, Chengdu, Sichuan 610075, China

In the article titled “The Effect of Acupuncture on the Motor Function and White Matter Microstructure in Ischemic Stroke Patients” [1], Ping Wu and Wenhua Huang contributed equally as corresponding authors.

## References

[1] Li Y., Wang Y., Zhang H., Wu P., Huang W. (2015). The effect of acupuncture on the motor function and white matter microstructure in ischemic stroke patients. *Evidence-Based Complementary and Alternative Medicine*.

